# The significance of single-cell transcriptome analysis in epididymis research

**DOI:** 10.3389/fcell.2024.1357370

**Published:** 2024-03-21

**Authors:** Meng-Meng Liu, Xin-Lei Feng, Chao Qi, Shu-Er Zhang, Guo-Liang Zhang

**Affiliations:** ^1^ College of Animal Science and Technology, Qingdao Agricultural University, Qingdao, Shandong, China; ^2^ Animal Products Quality and Safety Center of Shandong Province, Jinan, Shandong, China; ^3^ Provincial Animal Husbandry Station of Shandong Province, Jinan, Shandong, China

**Keywords:** epididymis, regional, cell composition, gene expression pattern, male fertility

## Abstract

As a crucial component of the male reproductive system, the epididymis plays multiple roles, including sperm storage and secretion of nutritive fluids for sperm development and maturation. The acquisition of fertilization capacity by sperm occurs during their transport through the epididymis. Compared with the testis, little has been realized about the importance of the epididymis. However, with the development of molecular biology and single-cell sequencing technology, the importance of the epididymis for male fertility should be reconsidered. Recent studies have revealed that different regions of the epididymis exhibit distinct functions and cell type compositions, which are likely determined by variations in gene expression patterns. In this research, we primarily focused on elucidating the cellular composition and region-specific gene expression patterns within different segments of the epididymis and provided detailed insights into epididymal function in male fertility.

## 1 Introduction

The epididymis, primarily composed of an epithelial membrane and a thin annular muscle, exhibits secretory functions that contribute to sperm nutrition and maturation. Species variations are observed in the length and morphology of the epididymis. Notably, mice possess an epididymal tube exceeding 1 m in length (approximately 100 times longer than the epididymis itself), while rats have 3 m and humans have 6 m (Von and Neuhaeuser, 1964; Turner et al., 1990; Jiang et al., 1994; Stoltenberg et al., 1998). As a component of the male reproductive tract, the development of the epididymis is dependent on androgens and originates from the Wolffian duct (Shaw and Renfree, 2014). Studies indicated that *Hoxa10* and *Hoxa11* genes, belonging to the homeobox (*Hox*) gene family, played crucial roles in epididymal development (Branford et al., 2000; Snyder et al., 2010).

The function of the epididymis is closely related to the sperm maturation. It has been established that mammalian spermatozoa undergo a process of “ripening” as they traverse the epididymis (Westfalewicz et al., 2017) While human sperm transport typically takes an average of 12 days (Rowley et al., 1970). In addition to these maturation steps, the epididymis provides a sophisticated microenvironment that allows for stationary storage of sperm during epididymal transit, avoiding premature sperm activation (Acott and Carr, 1984). Fundamentally, the four primary functions of the epididymis encompass storing, transporting, protecting, and eliminating sperm.

**FIGURE 1:**
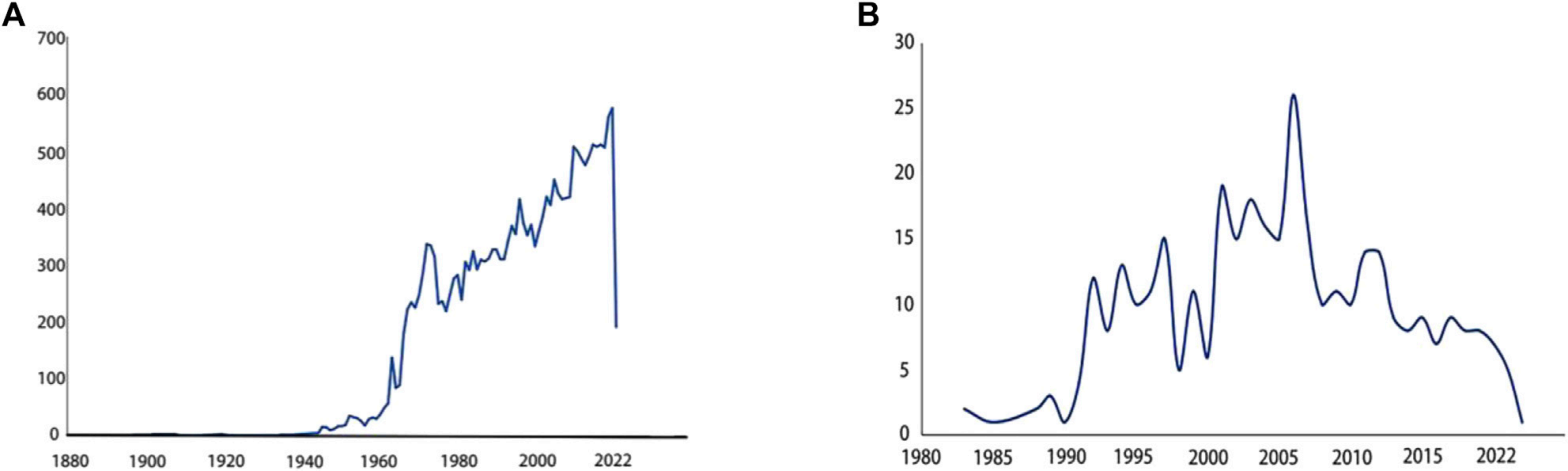
Paper counts published annually related to specific keywords **(A)** Paper counts published annually on epididymis. Search for epididymis keywords in PubMed. The number of research papers published each year is shown in the line graph. From 1960 to 2022, research papers about epididymis published in PubMed every year. More and more research papers are published every year, and the trend is rising in a straight line. **(B)** Paper counts published annually on region specific-gene expression in epididymis. Search for epididymis region specific gene expression keywords in PubMed. The number of research papers published each year is shown in the line graph. It is found that relevant research papers only appeared in 1983. From 1983 to 2022, the trend first rose and then declined.

The earliest report on the epididymis was published in the 18th century (National Library of Medicine, 1859): discutient application to the indurated epididymis, focusing on discutient application to treat indurated epididymis. Early understanding of the epididymis stemmed from various pathological studies (Rockwell, 1888; Bryant, 1892; Some Diseases of the Male Genital System, 1908) and investigations into its anatomical structures and functions (Griffiths, 1893; Watson, 1902). Since the 1950s, a significant number of research papers had emerged in this field. Over the past 70 years, extensive exploration had been conducted on different aspects of the epididymis. Initially, pioneers elucidated its anatomy in horses (Goglia, 1954), guinea pigs (Goglia and Magli, 1957) and humans (Montagna, 1952). Subsequently, cellular and chemical analyses gained prominence with cytochemical research and histochemical study of mouse epididymis (Allen, 1961; Allen and Slater, 1961; Birnbaum et al., 1961). Since the 1960s, continuous research efforts were dedicated to study the epididymis as evidenced by an increasing number of annual publications depicted in Figure 1A. With advancements in sequencing technology came a greater emphasis on understanding detailed functions of the epididymis. Consequently, its crucial role in sperm maturation was discovered (Salisbury et al., 1963; Salisbury and Graves, 1963). This led to further investigations into the proteome, transcriptome, and genetics of this organ resulting in numerous reported findings (Dacheux et al., 2009; Wong et al., 2020; Zheng et al., 2021). Transcriptomic and proteomic techniques facilitated progress in studying gene expression specific to the epididymis along with their associated functions (Lye and Hinton, 2004; Légaré and Sullivan, 2020). It is now well-established that each segment within this highly segmented structure expresses distinct genes as well as the related proteins (Domeniconi et al., 2016). Figure 1B illustrated that research pertaining to region-specific gene expression patterns began appearing since 1983 with an upward trend observed annually.

It had been reported that the environment of the epididymal duct might influence on the steps of sperm maturation (Shimazaki et al., 1976). During the process of mammalian sperm transport through the epididymis, the sperm encountered intraluminal fluid with disparate protein compositions, which interacted with the sperm surface and confers fertilization capabilities to the male gamete (Dacheux et al., 2005). Numerous studies had shown that a majority of sperm in the cauda region of epididymis were matured. As sperm passed through the epididymis, they undergo capacitation, acquire fertility, and could be used for *in vitro* fertilization (IVF) or artificial insemination (AI) (Axnér et al., 1999; Tsutsui et al., 2003; Toyonaga et al., 2011).

## 2 Relevant advancements Applied to research of epididymis

Rats and mice had been the most commonly used research models for studying the epididymis. In recent decades, advancements in technology, such as gene-knockout approaches (Eddy et al., 1996), transcriptome sequencing (Wu et al., 2021a), microarray analysis (Jervis and Robaire, 2003), proteomics (Fouchécourt et al., 2000), cell lines (Masuda et al., 2022), and single cell RNA-seq methods for transcriptome analysis (Rinaldi et al., 2020; Lang et al., 2022), had significantly enhanced our understanding of epididymal composition. Numerous knockout mouse models have exhibited phenotypes that impact male reproductive function. For instance, sperm from Clgn, Adam1a, Adam2, and Adam3 knockout mice were unable to migrate to the fallopian tubes (Cho et al., 1998; Ikawa et al., 2001; Nishimura et al., 2004). However, the dynamic functioning of these genes remained largely unknown. Both *in vivo* and *in vitro* models had been employed to investigate epididymal function. Currently, culturing techniques for epididymal cells had become well-established (Klinefelter et al., 1982; Chen et al., 1998; Lee et al., 2007). A rat caput epididymal cell line had demonstrated characteristics similar to epithelial principal cells found *in vivo* (Araki et al., 2002; Dufresne et al., 2005). The advent of RNA sequencing technologies had enabled researchers to analyze the transcriptomes of specific groups or even individual cells (Vazquez et al., 2019). Correlative studies had revealed novel distribution patterns of mitochondria and key genes that might be as-sociated with initial and subsequent sperm waves (Shi et al., 2021).

At present, there are many achievements in the application of single cell technology in the field of epididymis research. Human nasal, bronchial and epididymal epithelial cells were compared by single cell analysis. It has been found that when secretory cells, ciliated cells and basal cells are located in different anatomical locations, their transcriptome features are different (Paranjapye et al., 2022). Eight clusters of cell types have been identified in the proximal human epididymis, including secretory and basal epithelial cells, as well as rare clear cells, all of which have overlapping functions with bronchial and nasal epithelial cells (Paranjapye et al., 2022). Aged principal cells showed a variety of functional gene expression changes related to acrosome response and sperm maturation, suggesting that sperm activation and maturation is an asynchronous process during epididymal transport (Zhuang et al., 2023). At the same time, pathway alterations associated with aging were found in immune cells, particularly “cell chemotaxis” in Cx3cr1Hi epididymal dendritic cells (Zhuang et al., 2023). The monocyte specific expression of chemokine Ccl8 increased with age (Zhuang et al., 2023). Single-cell analysis of zebrafish testis revealed thousands of new marker genes specific to cell types, and through ligand receptor (LR) analysis, it was found that Zebrafish stromal cells had a stronger paracrine effect on germ cells than sertolith cells (Qian et al., 2022). The regulatory network, upstream transcription factors and downstream pathways of human epididymal fragment specific miRNA-mRNA were revealed, which provided a basis for further study of epididymal fragment specific function (Chen et al., 2023). Human epididymis was analyzed by single-cell RNA sequencing to further characterize efferent tubes and model systems (Leir et al., 2023).

The RNA-Seq dataset revealed a substantial number of genes expressed in different regions of the epididymis and the gene expression patterns exhibited significant differences among these regions. Notably, principal cells displayed diverse subsets that were distributed throughout the caput, corpus, and cauda of the epididymis (Rinaldi et al., 2020). However, numbers of genes could be used to distinguish the principal cells from different regions of the epididymal tube. RNase10, Cst11, Lcn2 and Mfge8 genes exhibited high expression levels in caput principal cells. Lcn5, Rnase9 and Plac8 genes were expressed in corpus principal cells (Rinaldi et al., 2020). Additionally, Gpx3, Hint1, Spink10 and Crisp1 showed significant expression in cauda principal cells (Rinaldi et al., 2020). Clear cells were identified by expression of the V-ATPase encoding marker genes such as Atp6v0c, Atpv1e1, and Atp6v1a (Rinaldi et al., 2020). Three clusters of basal cells were identified based on the shared expression of marker genes including Itga6 and Krt14 (Rinaldi et al., 2020). Another study employing single-cell analysis discovered eight subsets within the population of principal cells (Shi et al., 2021). According to GO analysis, two of these subsets were found to be enriched with genes associated with cilia organization and assembly, microtubule-based movement, and cilia-dependent cell movement (Shi et al., 2021). Other major subsets of epididymal epithelial cells were also examined. In detail, four cell subtypes were identified in cluster of basal cells, clear cells, and halo cells (Shi et al., 2021). In this study, the principal cells were characterized by the expression of Aqp9 and Cftr as marker genes, while Krt5 and Cldn1 served as marker genes for basal cells. Clear cells were identified by the presence of Atp6v1b1 and Foxil as marker genes (Shi et al., 2021). Additionally, novel region-specific genes were discovered in principal cells: Lcp1, Pemt, and Ucp2 exhibited high expression levels in the caput; Ccdc198, Ramp3, and Srgn showed high expression levels in the corpus; Atf3 and Zfp36 displayed high expression levels in the cauda of the epididymis (Shi et al., 2021). As new marker genes continue to be discovered, there are slight differences with marker genes in different cell types of different research. It was found that the number of mitochondria in the corpus and cauda of the mice was significantly higher than that in the caput of the epididymis (Shi et al., 2021). Mitochondria are a very important and abundant organelle type in the cytoplasm, involved in the production of adenosine triphosphate (ATP), the establishment of developmental capacity, the maintenance of calcium homeostasis, the regulation of apoptosis and other processes (Shi et al., 2021). The high mitochondrial count and MT% in the epididymis may be required to provide enough energy to synthesize and secrete thousands of molecules that are necessary for various sperm functions (Shi et al., 2021). Over the past 2 years, spatial transcriptomics had provided a wealth of quantitative gene expression data regarding mRNA distribution within tissue slices. These emerging technologies offered new opportunities for bioinformatics analyses in both research and diagnosis (Ståhl et al., 2016). In this paper, we provided an overview of various techniques utilized in epididymis research. Table 1 listed representative research papers showcasing applications of these new technologies within this field. It had been acknowledged that variations in gene and protein expression exist among distinct regions of the epididymis (Johnston et al., 2005), indicating diverse roles or functions in sperm maturation across these regions.

**TABLE 1:** Representative research papers on the application of new technologies in the field of epididymis.

Annual range	Representative research papers	New technology	Findings
1996–2000	Targeted disruption of the estrogen receptor gene in male mice causes alteration of spermatogenesis and infertility	Gene-knockout	To explore the effect of specific genes on epididymal function
2000–2001	Stallion epididymal fluid proteome: qualitative and quantitative characterization; secretion and dynamic changes of major proteins	Proteomics	Understand epididymal mRNA expression and protein secretion
2001–2005	Dynamic changes in gene expression along the rat epididymis	Microarray technology	Obtain differential expression profiles of epididymis multiple genes.
2005–2020	The mouse epididymal transcriptome: transcriptional profiling of segmental gene expression in the epididymis	Transcriptome sequencing technology	It aims to improve the integrity of epididymal transcriptome by using whole genome array, and provide higher sensitivity by studying fragments in each region
2020–2022	An atlas of cell types in the mouse epididymis and vas deferens	Single-cell RNA-Seq	A bird’s-eye view of the cell composition of epididymis was conducted to determine the new biological characteristics of epididymal cells.

## 3 Regional segmentation and functional differentiation in epididymis

**FIGURE 2:**
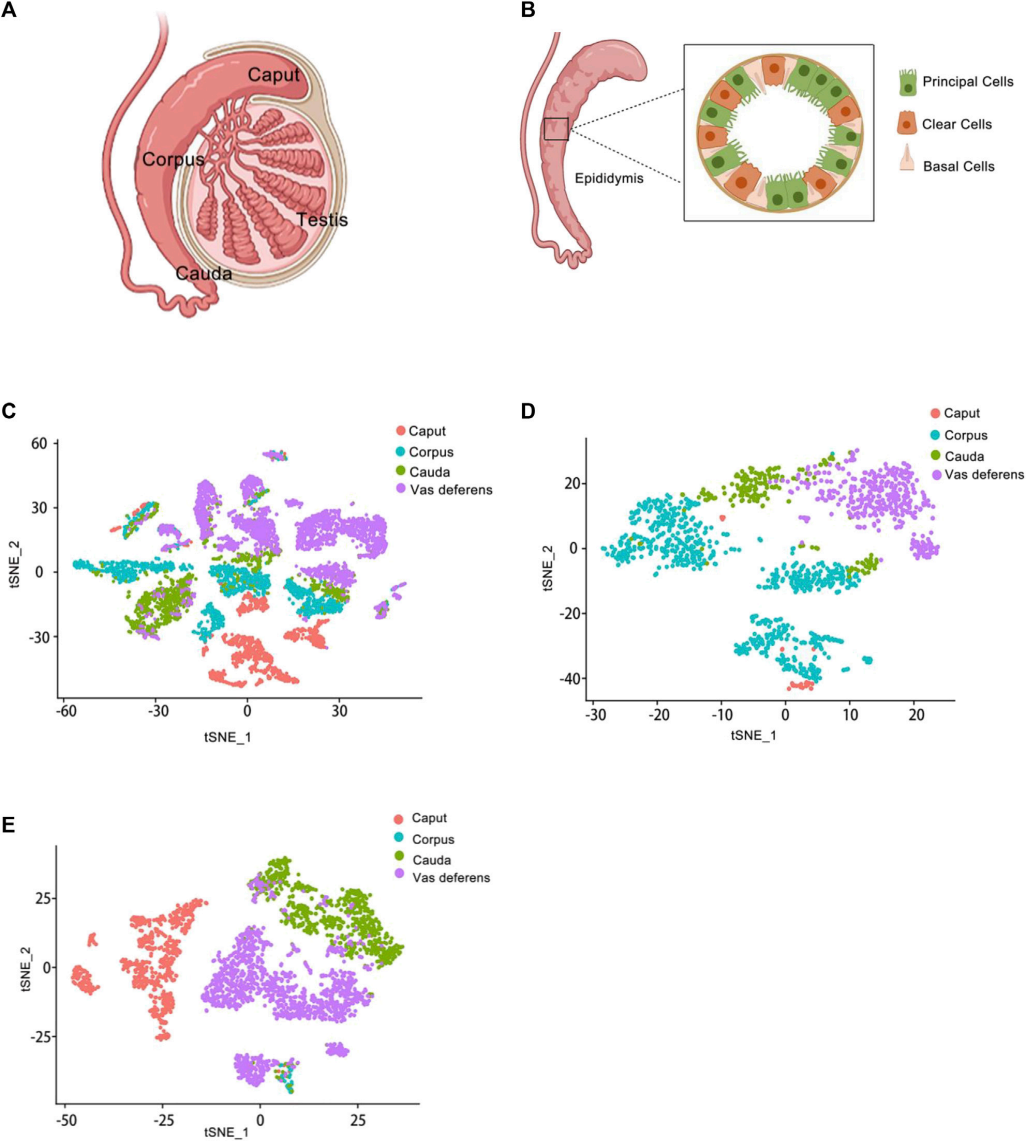
Epididymis atlas from anatomy to single cell level **(A)** Anatomy of the epididymis. The epididymis is attached to the testis. From top to bottom, it is the caput of the epididymis, the corpus of the epididymis, and the cauda of the epididymis. **(B)** Schematic representation of the main cell types in the epididymis. Several cell types make up the epididymal epithelium. It mainly consists of principal cells, basal cells and clear cells. Principal cells are the most abundant cells, which responsible for the absorption and secretion of substances into the epididymal cavity. Basal cells have a different morphological appearance, and they have the property of extending elongated body projections into the lumen between adjacent epithelial cells. Clear cells express proton-pumping ATPase in their apical membrane. **(C)** Single-cell cluster, clustered by t-SNE and annotated according to the four anatomical regions (caput, corpus, cauda and vas deferens). The data of epididymal caput, carpus, cauda and vas deferens were analyzed jointly. This is the t-SNE cluster diagram of four anatomical regions. Each color represents a cluster of cells in an anatomical region. The red dot represents the epididymis caput cells; The blue dot represents the epididymis corpus cells; The green dot represents the epididymis cauda cells; The purple dot represents the epididymis vas deference cells. **(D)** Reclustering of extracted basal cells, visualized by t-SNE and annotated according to the four anatomical regions. This figure shows the t-SNE cluster diagram of basal cells in the caput, carpus, cauda and vas deferens of epididymis regions. Each color represents a cluster of cells in an anatomical region. The red dots represent the epididymis caput basal cells; The blue dots represent the epididymis corpus basal cells; The green dots represent the epididymis cauda basal cells; The purple dots represent the epididymis vas deference basal cells. **(E)** Reclustering of extracted principal cells, visualized by t-SNE and annotated according to the four anatomical regions. This figure shows the t-SNE cluster diagram of principal cells in the caput, carpus, cauda and vas deferens of epididymis regions. Each color represents a cluster of cells in an anatomical region. The red dots represent the epididymis caput principal cells; The blue dots represent the epididymis corpus principal cells; The green dots represent the epididymis cauda principal cells; The purple dots represent the epididymis vas deference principal cells.

In previous studies, the epididymis had been traditionally classified into three regions–caput, corpus, and cauda. However, in rodents, the epididymis was further divided into four anatomical regions-initial segment, caput, corpus, and cauda (Gervasi and Visconti, 2017). While these divisions were well-established in rodents, they had not been clearly defined in the human epididymis. Previous reports had primarily used the caput, corpus, and cauda as boundaries for analyzing gene and protein expression patterns within the epididymis (Jervis and Robaire, 2001; Chauvin and Griswold, 2004). The caput, corpus, and cauda of the epididymis were proximal to distal, relative to the testis (Figure 2A).

Anatomical analysis of the human epididymis based on histology, proteomics data and 3D reconstruction had led to a subdivision of the caput, corpus and cauda into four six and ten regions respectively (Zhao et al., 2020). This research revealed significant variations in protein composition within different luminal regions of the epididymis (Dacheux et al., 2006). It should be noted that regional divisions of the epididymis also differ among other species. For instance, the mouse epididymis consisted of ten distinct segments while rats comprised fourteen segments (Adamali et al., 1999; Johnston et al., 2005). Furthermore, variations could be observed in size and shape of specific segments acrossing different species (Jelinsky et al., 2007).

The function of the epididymal corpus and cauda were found to be similar. Specifically, immune-related biological processes were enriched in both regions. During transport between the corpus and cauda of the epididymis, sperm acquired fertilization capacity. Currently, it had been demonstrated that sperm in the epididymal corpus area possess a similar ability to cauda sperm, including capacitation, oocyte fertilization, and survival during cryopreservation (Kunkitti et al., 2015; Kunkitti et al., 2016a; Kunkitti et al., 2016b). The cauda of the epididymis served as the final storage site for matured sperm prior to ejaculation (Souza et al., 2020). In many animal species, sperm could maintain fertility for several weeks within this environment (Esponda and Bedford, 1986). These findings highlighted the crucial role of processing and modification in each region of the epididymis for maintaining sperm function.

The biosynthesis of secreted proteins in the cauda of the epididymis, similar to that in the caput, was also regulated by androgen (Foldesy and Bedford, 1982; Wong et al., 1982; Esponda and Bedford, 1986; Regalado et al., 1993). In comparison to other mammals, human sperm production was relatively lower. Furthermore, the functional significance of temperature variation within the epididymis remained unknown (Bedford, 2015). In summary, sperm maturation was intricately linked to the unique regional functions within the epididymis, enabling successful fertilization.

## 4 Distinct cellular compositions across different epididymal regions

### 4.1 Epididymal epithelial and cellular phenotypes

In the past two decades, extensive research had been dedicated to comprehending the contribution of epididymal epithelial cells. The epididymal epithelium exhibited region-specific characteristics and functional diversity in the caput, corpus, and cauda regions. Each of these regions played a distinct role in ensuring that sperm acquired both motility and fertility functions. Furthermore, the epithelial lining primarily consisted of principal and basal cells (Arrighi, 2014). During the undifferentiated stage of epididymis development, small undifferentiated columnar cells formed the composition of epithelial cells (Sun and Flickinger, 1979). Subsequently, these undifferentiated short columnar cells differentiated into basal cells as well as high columnar/narrow cells which further develop into principal cells, clear cells, narrow cells, and apical cells (Dufresne et al., 2022).

Epididymal epithelial cells had been successfully isolated and cultured in many species, such as bull, mouse, rabbit, as well as human (Orgebin-Crist et al., 1984; Joshi, 1985; Moore and Hartman, 1986; Bongso and Trounson, 1996; Castellón and Huidobro, 1999). Several studies indicated that epithelial-mesenchymal interactions were important in epididymal morphogenesis (Higgins et al., 1989). The epididymal mesenchymal interactions, such as the phenotypic effects of androgens, were proved as the result of interactions between specific androgenic de-pending cells (De Gendt and Verhoeven, 2012). Besides, epididymal epithelium provided an optimal acidic liquid microenvironment in epididymal lumen (Hinton and Palladino, 1995; Zuo et al., 2011). Different regions of the epididymis contained a variety of epithelial cell types that served distinct functions.

Earlier studies had identified four types of epididymal epithelial cells: principal cells, narrow cells, clear cells, and basal cells (Jervis and Robaire, 2001). Subsequently, the classification was expanded to include six different cell types: principal cells, narrow cells, apical cells, clear cells, halo cells, and basal cells (Cornwall, 2009). Furthermore, numerous studies had categorized epididymal epithelial cells into five types: principal cell, narrow cell, clear cell, apical cell and basal cell (Shum et al., 2009). The presence of various cell types varies across species; for instance, apical cells, narrow cells, clear cells, and halo cells (Arrighi, 2014). Recently, single cell RNA-sequencing samples had enabled the identification of multiple subtypes within the epididymis (Leir et al., 2020; Rinaldi et al., 2020). In 2020, the study by Leir et al. (Leir et al., 2020) obtained 1876 cells, 1309 cells, and 2114 cells from human epididymis aged 32, 57 and 32 years, respectively. Eight distinct proximal epididymal cell types were identified in humans including principal cells, basal cells, clear cells, stomal cells, apical/narrow cells, immune cells, sperm, and efferent ducts (Leir et al., 2020). The identification of specific marker genes allowed for the division of each cell type into multiple subtypes. In the study, a total of nine principal cell sub-types and three basal cell subtypes were analyzed from the dataset (Rinaldi et al., 2020). Further investigation would be required to elucidate the contribution of different epididymal epithelial cell subtypes to sperm maturation.

### 4.2 Principal cells

The most abundant epididymis epithelial cells were the principal cells, which dis-tributed throughout the whole epididymis. According to the analysis of epididymis, the principal cells accounted from 65% to 80% of the epididymal epithelium. It is reported that the number of principal cells was at least three times more than the sum of other cell types (Hermo et al., 1994; Joseph et al., 2011). The cells were mainly responsible for the absorption and the secretion of substances into the epididymal cavity, so it had significantly secretory activity (Cornwall, 2009). In addition, the principal cells were the places where liquid, ions, antioxidants and exons were produced and released (Sullivan and Saez, 2013).

The epididymal junction complex between adjacent principal cells comprised apical adhesions and tight junctions, leading to the formation of the blood-epididymal barrier (Cyr et al., 1995). The blood-epididymis barrier limited the molecular exchange between blood and lumen, providing a guarantee for the stability of the epididymal lumen environment. Consequently, specific microenvironments were established within the epididymal lumen, which played a crucial role in sperm maturation. Principal cells secreted proteins that bound to maturing spermatozoa and regulated their maturational process (Cyr et al., 2007). Dysfunction of epididymis principal cells could potentially impact sperm motility quality and fertilization capacity. Notably, Occludin (OCLN), a tight junction protein, localized at the apical junction of proximal epididymis epithelium’s principal cells. Deletion mutation of OCLN had been shown to cause male infertility in mice (Liu et al., 2022a).

### 4.3 Basal cells

Basal cells were present in the columnar pseudostratified epithelium that covered the mammalian epididymis (Arrighi, 2014). These cells could be found in the epididymal epithelium of all species, including caput, corpus, and cauda regions (Hermo et al., 1994; Arrighi, 2014). These cells were located in the basal layer and extend long and slender cytoplasmic projections to the lumen. It was generally believed that there were more basal cells in the epididymal corpus and fewer in the cauda (Arrighi et al., 1991; Schon and Blottner, 2009). Initially, basal cells were thought to have macrophage-like qualities and were associated with epididymal immunity (Yeung et al., 1994; Seiler et al., 1998). Basal cells might scan and perceive the lumen environment of pseudostratified epithelium and regulate epithelial function through the mechanism of crosstalk with other epithelial cells (Shum et al., 2008). In addition to their role in regulating epithelial function through crosstalk with other cell types, basal cells also had a capacity to monitor the fluid environment within the epididymis.

Early studies showed that basal cells of epididymis could be detected as stem cells (Mandon et al., 2015; Mou et al., 2016). Furthermore, several signaling pathways related to stem cell functions such as Notch1, Hedgehog, p63, and Wnt were enriched in basal cells (Yalcin-Ozuysal et al., 2010), some of which were known to be involved in sperm function. Specifically, Wnt ligands had been shown to promote stability of sperm proteome and enhanced sperm motility by orchestrating post-transcriptional sperm maturation programs (Koch et al., 2015). The relationship between stem cell-related signaling pathways within basal cells and sperm function warranted further investigation. Primary cilia were observed on both surfaces of basal cells as well as within intercellular spaces. Damage to these primary cilia on basal cells lead to imbalanced apoptosis rates among epididymal epithelial cells, thus highlighting their role as guardians for maintaining homeostasis within pseudostratified epithelia (Girardet et al., 2022).

### 4.4 Other cells

Apical cells were mainly located in the initial segment of the epididymal epithelium and had endocytosis activity. Narrow cells also existed only within the initial segment, as their name suggested, being narrower than the adjacent principal cells. Both of apical cells and narrow cells had been shown to secrete H+ into the epididymal lumen and are responsible for endocytosis (Cornwall, 2009). Clear cells were large endocytotic cells scattered among the principal cells in the caput, corpus and cauda regions. In addition to secretion, endocytosis of lumen proteins was the main function of epithelial clear cells (Moore and Bedford, 1979). Narrow, apical and clear cells were strongly expressing vacuolar proton pump ATPase (V-ATPase) in their apical membranes, which was considered to be the reason for proton secretion into the lumen (Da Silva et al., 2007; Kujala et al., 2007; Belleannée et al., 2010).

In the epididymis, intraepithelial cells were believed to perform distinct and integrated functions. Recent studies had demonstrated that basal cells regulated electrolyte transport in principal cells by releasing paracrine factors (Cheung et al., 2005). It was concluded that cell–cell interaction underlied the formation of the epididymal environment in maturing spermatozoa. Therefore, the intricate interplay between different epithelial cell types contributed to the activation of luminal acidification in the epididymis, which was essential for sperm maturation and storage.

## 5 Different gene expression patterns in different epididymis regions

### 5.1 Specially expressed genes and gene families

Regional specificity of gene expression in epididymis had been proposed for a long time (Douglass et al., 1991; Sullivan et al., 2011). Some genes were predominantly expressed in the epididymis, which suggested that these genes might possess epididymis-specific functions. To gain insights into this phenomenon, the mRNA of the caput, corpus and cauda of the epididymis had been sequenced by transcriptome sequencing. Specific mRNA synthesis supported the concept that the region-specific expression pattern of epididymal tubule transcription was the main molecular basis of the region-specific expression pattern of tubule lumen proteins (Douglass et al., 1991). Furthermore, each gene had distinct spatial expression and regulation in the epididymis. The genes expressed in the epididymis played an important role in sperm maturation and motility, and changed in epididymal-specific gene expression may lead to infertility. In the following, we would specify the expression of representative epididymal genes.

The mouse epididymis transcripts were analyzed based on a 17,000 oligonucleotides microarray. These genes were up or downregulated more than four times between at least two different fragments of mouse epididymis (Ståhl et al., 2016). The expression patterns of these genes identify distinct patterns of segmental regulation. Analysis based on rat microarray technology found that the number of genes detected in each region of the epididymis varied greatly. Notably, 53% of the genes with the highest expression were expressed in the cauda (626 out of 1176 genes) (Jervis and Robaire, 2001). Recently, the region-specific ex-pression genes in sheep were analyzed by GO and KEGG analysis to screen the key genes related to sperm maturation. A total of 129, 54 and 99 specific genes were obtained in the caput, corpus and cauda, respectively (Wu et al., 2021a). The heatmap showed that six genes of the *LCN* family (the lipocalin (*Lcn*) family was a hydrophobic ligand binding protein) were highly expressed in the sheep epididymal caput. *RNase10* (ribonuclease, RNase A family, 10, predicted to enable nucleic acid binding activity) was also highly specifically expressed in caput. It was the same for other species for which *RNase10* had been identified, such as humans, mice and pigs (Castella et al., 2004; Devor et al., 2004; Krutskikh et al., 2012).

### 5.2 scRNA-seq showed gene expression patterns in the epididymis

We reanalyzed the data provided in previous research (Rinaldi et al., 2020) with the Seurat software package, collecting the data of the mouse epididymal caput, corpus, cauda and vas deferens. The organ was divided into four regions: caput, corpus, cauda, and vas deferens. The single-cell RNA sequencing library was prepared using the Chrome single-cell 3′kit V2 (10X Genomics). Using single-cell data uploaded to the GEO (Gene Expression Omnibus) website combined with our own mouse epididymis data, we visualized the expression of several genes in basal cells and principal cells.

**FIGURE 3:**
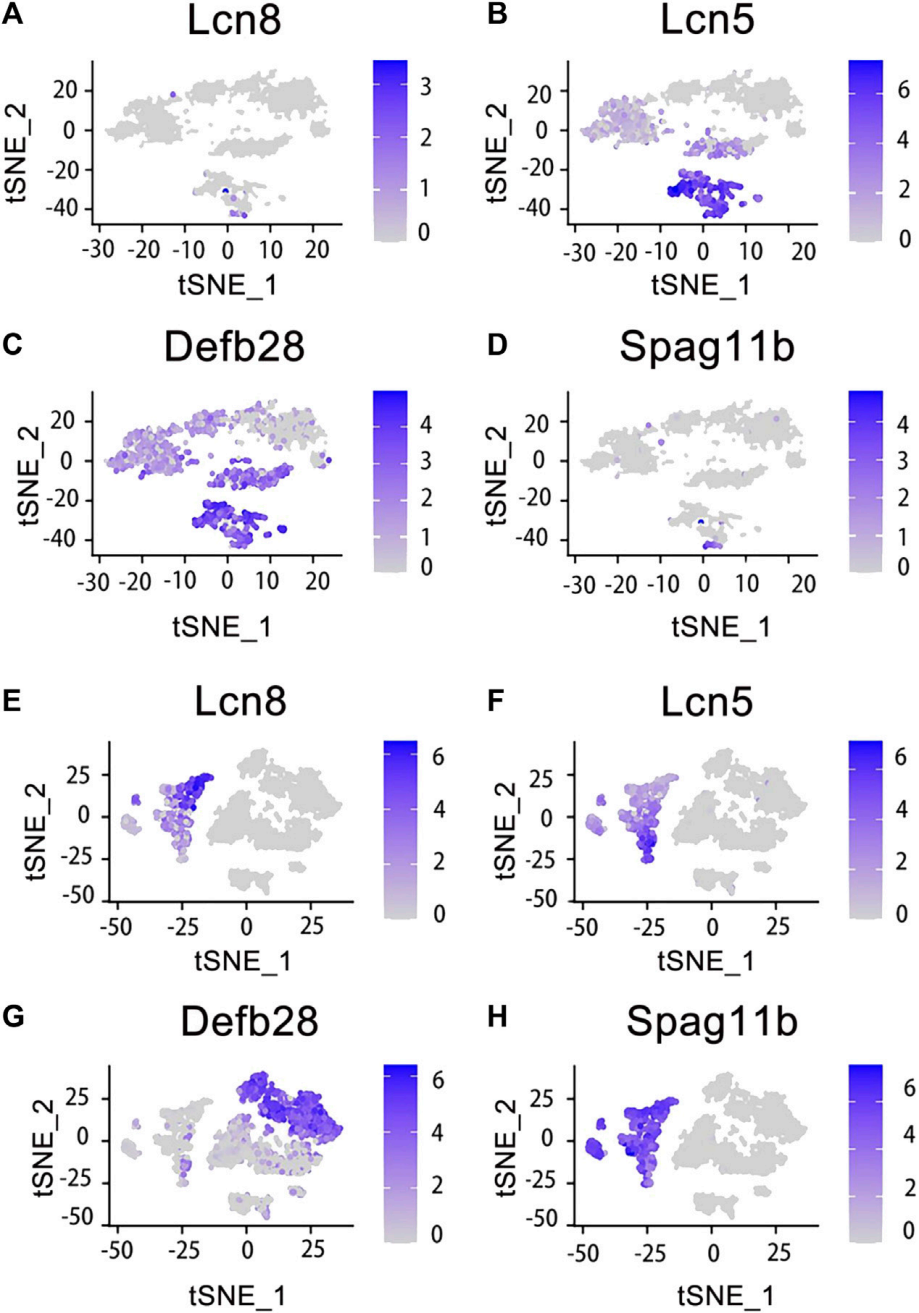
Expression of specific genes across basal cells and principal cells. Those genes were selected and looked at their epididymal distribution in different parts respectively. The tSNE cluster map shows the differential expression of genes in basal cells. The darker blue indicates the higher expression level. **(A)** *Lcn8* is highly expressed in epididymis caput basal cells. **(B)** *Lcn5* is highly expressed in epididymis caput and corpus basal cells. **(C)** *Defb28* is highly expressed in corpus, cauda and vas deferens of epididymis basal cells. **(D)** *Spag11b* is highly expressed in epididymis caput basal cells. **(E)** *Lcn8* is highly expressed in epididymis caput principal cells. **(F)** *Lcn5* is highly expressed in epididymis caput principal cells. **(G)** *Defb28* is highly expressed in epididymis cauda principal cells. **(H)** *Spag11b* is highly expressed in epididymis caput principal cells.

Finally, we obtained a total of 9167 individual cells in a single assay (Figure 2C). Within the dataset, 1619 cells were collected from the caput of the epididymis, 2345 cells from the corpus of the epididymis, 1658 cells from the cauda of the epididymis, and up to 3545 cells were collected from the vas deferens. To visualize these four regions of data, we employed t-distributed stochastic neighbor embedding (t-SNE), a dimensionality reduction technique that enables visualization of data pointed on a two-dimensional map. In the t-SNE map, genes with similar functions tended to cluster together and form distinct groups within a coordinate system. Each region of the epididymal sample is represented by a different color (Figure 2C). Subsequently, principal cell and basal cell populations representing two major cell types in the epididymis were individually extracted and clustered. By employing t-SNE analysis and annotating them based on their respective regions, we successfully identified basal cells (Figure 2D) and principal cells (Figure 2E). There were 1410 cells within the basal cluster (Figure 2D) and 2099 cells in the principal cluster (Figure 2E). Furthermore, numerous genes were selected for examination regarding their distribution across different regions within the epididymis (Figure 3).

## 6 Discussion

The epididymis comprises convoluted tubules that connect the testis and vas deferens within the male reproductive system. It plays a pivotal role in regulating sperm maturation and fertilization processes. Sperm maturation necessitated epididymal processing, encompassing regional alterations in spermatozoa structure and molecular composition (Jones et al., 2007). Additionally, it involves immune and metabolic physiological mechanisms. Notably, numerous macrophages were present within the epididymal lumen and were capable of recognizing and eliminating suboptimal spermatozoa. Consequently, the epididymis assumed a critical function in both sperm maturation and the acquisition of fertilization competence (Elbashir et al., 2021).

With the advancement of sequencing technologies (Wong et al., 2020; Wu et al., 2021a; Zhao et al., 2021a; Liu et al., 2022b), a greater understanding of the molecular mechanisms underlying epididymal function has been achieved. The significance of the epididymis in male reproductive biology has garnered increasing attention. Furthermore, distinct regional disparities within the epididymis have been elucidated (Belleannée et al., 2012; Cyr et al., 2018; Wu et al., 2021b; Zhao et al., 2021b). For instance, the caput region exhibited heightened activity and accounted for approximately 70%–80% of total protein secretion (Cornwall, 2009). The corpus of the epididymis plays a pivotal role in immune functions. The comprehension of the epididymis evolved from its initial morphological and structural characterization to encompass diverse functional aspects across different regions (Martin et al., 1902; Sullivan et al., 2019; James et al., 2020; Zhao et al., 2020). Notably, unique gene expression patterns were identified in the caput, corpus, and cauda regions through techniques such as microarray or single-cell RNA sequencing analysis. In summary, while the caput region secretes nutrients and androgens to facilitate sperm maturation, sperm traverse through the corpus region towards the cauda, which acts as a barrier against autoimmune reactions, additionally serving as a conduit for transporting sperm to the vas deferens. Consequently, functional discrepancies exist among various regions within the epididymis due to differential gene expression profiles that drive specific functions associated with each respective region.

The male reproductive system exhibited significant interspecies variability. In the epididymis of rats and mice, distinct regions displayed pronounced differences (Abou-Haïla and Fain-Maurel, 1984). Due to their shorter life cycle than humans, rodents have become the most common animal model in research. Recently, dogs have emerged as viable biological models for studying the molecular functions of the epididymis (Kirchhoff, 2002). However, extrapolating reproductive characteristics of other mammals based solely on a specific species might lead to biased conclusions in terms of reproductive biology.

The cell classifications of the epididymal epithelium in each region of the epididymis varied (Chen et al., 2021). Among them, the principal cells constituted 80% of the epithelial cells in the epididymis tubule. Further analysis revealed distinct gene expression patterns for the same cell type across different regions of the epididymis. In this study, we reanalyzed and visualized gene expression data from the mouse epididymis and vas deferens based on previous findings and publicly available single-cell datasets. Marker genes specific to caput principal cells included *RNase10*, *Cst11*, and *Lcn2*. Similarly, the *Lcn5*, *RNase9*, and *Plac8* genes distinguished corpus principal cells, while the *Gpx3*, *Klk1b27*, *Hint1*, and *Gstm2* genes characterized cauda principal cells. Basal cells were identified by the *Itga6* and *Krt14* genes. Interestingly, our analysis revealed differential mRNA expression levels between principal and basal cells for several genes, including *Lcn5,* which showed high expression in caput principal cells but was significantly detected in both caput and corpus basal cells; *Defb28* exhibited high expression in cauda principal cells but was significantly detected in epididymis basal cells as well (Rinaldi et al., 2020). The results demonstrated the essential role of *Rnase10* in sperm adhesion within the epididymis, a critical characteristic for mouse sperm transport in the female vagina (Krutskikh et al., 2012). Combined with the data analysis on *Lcn5* in the principal cells, we indicated that the caput of the mouse epididymis was related to lipid metabolism and adhesion. Similarly, basal cells exhibited significant enrichment of genes related to membrane transport and lipid metabolism (Rinaldi et al., 2020). These findings suggested that different cell types share similarities, contributing to an optimal environment for sperm maturation within the epididymal cavity. Additionally, certain coregulatory factors displayed fragment-specific enrichment patterns during their expression processes, suggesting their potential contribution to specific responses (Browne et al., 2016). Each region of the epididymis possesses a unique transcriptomic signature, which regulates luminal composition and ultimately influences male sperm maturation.

## 7 Conclusion

The intricate patterns of gene expression and cellular composition in the caput, corpus, and cauda regions of the epididymis contribute to distinct functions in sperm maturation. However, interspecies variations exist in gene expression within the epididymis. Exploring the functional diversity across different regions of the epididymis presents an exciting avenue for research. It is anticipated that advancements in sequencing technologies, such as spatial transcriptomics, would play a pivotal role in unraveling the complexities of epididymal biology.

## Data Availability

The datasets presented in this study can be found in online repositories. The names of the repository/repositories and accession number(s) can be found in the article/supplementary material.

## Abbreviations

AI, artificial insemination; DEFB, defensin, beta; Hox, homeobox; IVF, in vitro fertilization; LCN, lipocalin; OCLN, occludin; Rnase10, ribonuclease A family member 10 (inactive); t-SNE, t-distributed stochastic neighbor embedding.
